# Rapid detection of *Heterobasidion annosum* using a loop-mediated isothermal amplification assay

**DOI:** 10.3389/fcimb.2023.1134921

**Published:** 2023-04-28

**Authors:** Zhou Hong-min, Yu Jian, Liu Ying, Yuan Yuan, Wu Cui-ping, Dai Yu-cheng, Chen Jia-jia

**Affiliations:** ^1^ Institute of Microbiology, School of Ecology and Nature Conservation, Beijing Forestry University, Beijing, China; ^2^ College of Landscape Architecture, Jiangsu Vocational College of Agriculture and Forestry, Zhenjiang, China; ^3^ Animal, Plant and Food Inspection Center, Nanjing Customs, Nanjing, China

**Keywords:** *Heterobasidion annosum*, pathogens of *Pinus*, LAMP assay, molecular diagnosis, port quarantine

## Abstract

*Heterobasidion annosum* is one of the most aggressive pathogens of *Pinus* forests in Europe, causing considerable economic losses. To detect *H. annosum* for disease diagnosis and control, we developed a loop-mediated isothermal amplification (LAMP) reaction with a primer set designed from the glyceraldehyde 3-phosphate dehydrogenase (GAPDH) DNA sequences of *H. annosum*. In our study, this LAMP assay was found to be capable of efficiently amplifying the target gene within 60 min at 63°C. In specificity tests, *H. annosum* was positively detected, and other species were negative. The detection limit of this assay was found to be 100 pg·μL^-1^, and the assay was also successfully tested for use with basidiospore suspensions and wood samples. This study provides a rapid method for diagnosing root and butt rot caused by *H. annosum*, which will be of use in port surveillance of logs imported from Europe.

## Introduction

*Heterobasidion annosum* (Fr.) Bref. sensu lato (s.l.) has been studied intensively over several decades, with interfertility studies showing that *H. annosum* s.l. is a species complex (Korhonen, 1978; Dai and Korhonen, 1999; Dai et al., 2003). Recently, species of *Heterobasidion* have been divided into three groups using multilocus phylogenetic approaches; furthermore, the pathogenic species *H. annosum* sensu stricto (s.s.) has been found to be a sister to *H. irregulare* Garbel. & Otrosina. Most taxa of *H. annosum* s.l. are distributed in the conifer forests of the northern hemisphere (Chen et al., 2015; Dai et al., 2021a, b; Yuan et al., 2021; Wu et al., 2022).

*Heterobasidion annosum* is one of the most aggressive pathogens in the destruction of pine plantations in Europe (Edmonds et al., 1989; Woodward et al., 1998; Dai and Korhonen, 1999). The root and butt rot caused by *Heterobasidion* s.l. species can destroy the most valuable part of the tree (Korhonen and Stenlid, 1998; Niemelä and Korhonen, 1998; Seifert, 2007), depreciating the usability of the timber (Aza et al., 2021) and lowering the tree’s resistance to strong winds (Oliva et al., 2008). Furthermore, *H*. *annosum* s.l. may remain active in residual stumps and roots for decades until the next rotation (Rishbeth, 1951; Greig and Pratt, 1976). Significantly, *H. annosum* s.l. grows more quickly in dead trees than in living trees (Bendz-Hellgren et al., 1999). Hence, poor thinning and logging operations may increase the incidence of annosum-related rot (Shaw et al., 1995; Morrison and Johnson, 1999). Dai et al. (2021a) proposed that the most aggressive conifer pathogens, *H*. *abietinum*, *H*. *annosum* s. s., *H*. *irregulare*, *H*. *occidentale*, and *H*. *parviporum*, should be identified as quarantine fungi, as they are not found in China. Therefore, effective detection of annosum-related rot is important.

Over the past several decades, various methods for *H*. *annosum* detection have been developed, mainly focusing on morphological characters, mating tests, and molecular strategies. Traditionally, morphological identification of *H. annosum* has relied on macroscopic and microscopic observations (Rishbeth, 1951; Greig and Pratt, 1976; Tokuda et al., 2009; Aberg et al., 2016). However, once the basidiomata can be observed, it is already too late to protect the trees in question from decay (Garbelotto and Gonthier, 2013). Although mating tests are a relatively reliable method to determine compatibility with known species, they take time (Korhonen, 1978; Mitchelson and Korhonen, 1998; Dai and Korhonen, 1999; Dai et al., 2002). In fact, as all the classical diagnostic methods are complicated, time-consuming, and require professional skill, researchers have been investigating molecular methods of assay. A potential polymerase chain reaction (PCR) method offers great promise for detection of pathogenic fungi because of its speed and specificity (Schulze, 1999). Multiplex real-time PCR assay, with better resolution than traditional technology, has already been conducted by several researchers (Hietala et al., 2003; Ioos et al., 2019), and qPCR technology has been used to measure the distribution of species of *Heterobasidion* (Oliva et al., 2017).

Although PCR technology has already been applied in detection of *H. annosum* due to its sensitivity and specificity, long periods of time and expensive laboratory instruments are still required for these procedures. These intrinsic disadvantages prevent this method from being used in resource-limited regions. Loop-mediated isothermal amplification (LAMP) is an alternative method that amplifies target DNA sequences with high sensitivity and specificity under isothermal conditions (Notomi et al., 2000). The technology has previously been applied in pathogen detection (Sillo et al., 2017; Kong et al., 2020; Vettraino et al., 2021). So far, LAMP technology has been widely used in the medical field (Parida et al., 2005; Parida et al., 2007; Santiago, 2021), food science (Petersen et al., 2021), and plant protection (Franco Ortega et al., 2019; Enicks et al., 2020). The North American species *H. irregulare* Garbel. & Otrosina was detected by LAMP using a HirrSC3 gene within cytochrome P450 monooxygenase (Sillo et al., 2017). However, methods for rapid detection of *H. annosum* have rarely been reported.

LAMP utilizes a *Bst* DNA polymerase with stand-displacement activity, along with two inner primers (FIP, BIP) and two outer primers (F3, B3) that recognize six separate regions within a target DNA sequence (Notomi et al., 2000). Correct recognition of all six regions by the primers ensures the specificity of the assay. Positive reactions can be examined in the products, as follows: turbidity of magnesium phosphate increases (Mori et al., 2001); ladder-like bands can be observed on gel electrophoresis; and color changes can be induced in the reaction system through the addition of DNA-intercalating dyes (Goto et al., 2009). The metal ion hydroxynaphthol blue (HNB) is a reliable indicator of DNA amplification because of the low risk of cross-contamination along with sensitivity equivalent to that of SYBR green, and the results can easily be judged with the naked eye (Goto et al., 2009).

In this study, we aimed to develop a simple LAMP detection method for specific identification of *H. annosum* and to evaluate its accuracy in detecting wood decay caused by *H. annosum*.

## Materials and methods

### Culture conditions and DNA extraction

This study used forty-five cultures and specimens which were maintained at the Institute of Microbiology, the Beijing Forestry University (BJFC, Beijing, P.R. China), Jiangsu Vocational College of Agriculture and Forestry (JSAFC), and the Natural Resources Institute, Finland (Luke, Helsinki, Finland) (**Table 1**). Fungal strains were cultured on potato dextrose agar (PDA) (Caten and Jinks, 1968; Gams et al., 1998) in 90 mm petri dishes at 25°C for 28 days. In order to obtain abundant mycelia, fungal strains were cultured on potato dextrose agar for 7 days.

**Table 1:** Fungal isolates and basidiomata used in this study.

Species	Specimen No.	Host	Location	Result	GenBank accession		Reference or GenBank accessions
					ITS	GAPDH	
*Albatrellus alpinus*	Cui 17023	On ground in forest of *Pinus* sp. and *Quercus* sp.	China	–	MW534154	—	Zhou et al., 2021
*Aleurocystidiellum disciformis*	He 3159	*Quercus* sp.	China	–	KU559340	—	Liu et al., 2017
*Aleurodiscus amorphus*	Ghobad-Nejhad2464	*Abies* sp.	China	–	KU559342	—	Liu et al., 2017
*Amylonotus labyrinthinus*	Yuan 1475	Angiosperm	China	–	KM107860	—	Liu et al., 2017
*Amylosporus succulentus*	Dai 7802	Lawn	China	–	KM213669	—	Liu et al., 2017
*Amylostereum orientale*	He 479	*Cunninghamia lanceolata*	China	–	JX049987	—	Liu et al., 2017
*Bondarzewia submesenterica*	Cui 10345	*Podocarpus* sp.	China	–	KJ583204	—	Chen et al., 2016
*Bondarzewia podocarpi*	Cui 6380	*Podocarpus*	China	–	KJ583206	—	Chen et al., 2016
*Dentipellis coniferarum*	Cui 10063	*Abies* sp.	China	–	JQ349106	—	Chen et al., 2016
*Echinodontium japonicum*	Dai 7378	Angiosperm	China	–	KY172887	—	Liu et al., 2017
*Echinodontium tinctorium*	HHB 12866-Sp	*Tsuga* sp.	USA	–	KY172888	—	Liu et al., 2017
*Heterobasidion annosum*	06071/1	*Pinus pinea*	Italy	+	—	KJ651761	Chen et al., 2015
*Heterobasidion annosum*	06125/2	*Pinus sylvestris*	Russia	+	—	KJ651762	Chen et al., 2015
*Heterobasidion annosum*	06129/6	*Pinus sylvestris*	Russia	+	—	KJ651763	Chen et al., 2015
*Heterobasidion annosum*	09001/1	*Pinus* sp.	Italy	+	—	KJ651765	Chen et al., 2015
*Heterobasidion annosum*	93691/6	—	England	+	—	KJ651760	Chen et al., 2015
*Heterobasidion annosum*	Dai 6540	*Pinus* sp.	Italy	+	—	—	Chen, 2015
*Heterobasidion annosum*	Dai 14857	*Pinus* sp.	Poland	+	—	—	Chen, 2015
*Heterobasidion abietinum*	00051/1	*Picea* sp.	Italy	–	—	AJG42512	Chen et al., 2015
*Heterobasidion amyloideum*	L 1878	Gymnosperm	China	–	—	KJ651758	Chen et al., 2015
*Heterobasidion araucariae*	65008	*Araucaria* sp.	Australia	–	—	KJ651766	Chen et al., 2015
*Heterobasidion armandii*	Dai 17605	*Pinus* sp.	China	–	MT146482	—	Yuan et al., 2021
*Heterobasidion australe*	Y 05054/1	Gymnosperm	China	–	—	—	Chen, 2015
*Heterobasidion insulare*	Dai 15095	*Pinus* sp.	China	–	—	MT157728	Yuan et al., 2021
*Heterobasidion irregulare*	01056	*Tsuga* sp.	Canada	–	—	KJ651780	Chen et al., 2015
*Heterobasidion linzhiense*	Dai 5408	*Abies* sp.	China	–	—	KJ651788	Chen et al., 2015
*Heterobasidion occidentale*	79034/VE	—	USA	–	—	AJG42548	Chen et al., 2015
*Heterobasidion orientale*	N 97011/7	—	China	–	—	KJ651794	Chen et al., 2015
*Heterobasidion parviporum*	04121/3	*Picea* sp.	Finland	–	—	KJ651800	Chen et al., 2015
*Heterobasidion subinsulare*	Li 140804-30	*Pinus* sp.	China	–	—	MT157733	Yuan et al., 2021
*Heterobasidion subparviporum*	Cui 6961	*Larix* sp.	China	–	—	KJ651809	Yuan et al., 2021
*Heterobasidion tibeticum*	I 04031/1	Gymnosperm	China	–	—	KJ651810	Chen et al., 2015
*Larssoniporia incrustatocystidiata*	Dai 13607	Angiosperm	China	–	KM107863	—	Liu et al., 2017
*Laurilia sulcata*	He 20120916-7	*Abies* sp.	China	–	KY172894	—	Liu et al., 2017
*Lauriliella taxodii*	FP-105464-Sp	*Taxodium distichum*	USA	–	KY172896	—	Liu et al., 2017
*Peniophora erikssonii*	Cui 11871	—	China	–	MK588771	—	Xu et al., 2023
*Peniophora albobadia*	He 2159	—	USA	–	MK588755	—	Xu et al., 2023
*Peniophora bicornis*	He3609	—	China	–	MK588763	—	Xu et al., 2023
*Peniophora crassitunicata*	He 3814	—	China	–	MK588770	—	Xu et al., 2023
*Peniophora vietnamensis*	He 5242	—	Vietnam	–	MK588760	—	Xu et al., 2023
*Peniophora yunnanensi*	CLZhao3978	—	China	–	OP380617	—	Xu et al., 2023
*Perplexostereum endocrocinum*	Dai 15998	Gymnosperm	China	–	KY172899	—	Liu et al., 2017
*Pseudowrightoporia japonica*	Dai 12086	Angiosperm	China	–	KJ513293	—	Chen, 2015
*Wrightoporia subavellanea*	Dai 11484	*Pinus*	China	–	KJ513295	—	Chen, 2015
*Wrightoporiopsis amylohypha*	Yuan 3460	Angiosperm	China	–	KM107875	—	Chen, 2015

Mycelia and basidiomata were ground in liquid nitrogen and subsequently collected in 1.5-mL microfuge tubes. Genomic DNA was extracted using the CTAB rapid plant genome extraction kit (Aidlab Biotechnologies Co., Ltd., Beijing, China) according to the manufacturer’s instructions, with some modifications (Chen et al., 2015). The concentration of the extracted DNA was evaluated using a Nanodrop spectrophotometer (Thermo Fisher Scientific, USA) following Kong et al. (2020); this was then diluted in 10-fold serial dilutions to produce concentrations from 10 ng·μL^-1^ to 10 fg·μL^-1^ and stored at –20°C. The cultures and specimens used were identified by morphological examination, and/or by sequencing Glyceraldehyde-3-phosphate dehydrogenase (GAPDH) (Chen et al., 2015) or the internal transcribed spacer (ITS) (**Table 1**).

### Optimization of the LAMP reaction

The LAMP reaction was performed according to previously described methods (Niu et al., 2012; Duan et al., 2014; Kong et al., 2020; Vettraino et al., 2021). The final LAMP reaction (26 μL volume) was performed by combining 2.5 μL 10 × ThermoPol buffer, 1.6 μmol·L^-1^ forward inner primer (FIP) and backward inner primer (BIP), 0.2 μmol·L^-1^ B3 and F3 primers, 0.8 μmol·L^-1^ LB and LF primers, 5 mmol·L^-1^ Mg^2+^, 0.8 mol·L^-1^ betaine, 1.4 mmol·L^-1^ dNTPs, 300 μmol·L^-1^ HNB, 8 U of *Bst* DNA polymerase, and 2 μL DNA template.

The LAMP reaction mixtures were heated at a range of reaction temperatures (viz., 61°C, 62°C, 63°C, 64°C, and 65°C) for 60 min to select the optimal temperature (**Figure S1**). Additionally, LAMP reactions were performed at the optimal reaction temperature (63°C) for 15 min, 30 min, 60 min, and 90 min in order to select the shortest viable reaction time (**Figure S2**). Runs were performed with positive controls (*H. annosum*), negative controls (14 *Heterobasidion* spp. and 24 other fungi), and controls consisting of distilled water without DNA. The assays were evaluated by observation of the HNB color change from violet to blue, which denotes positive amplification, while a negative assay remains violet. The optimum temperature and shortest viable time were identified as 63°C for 60 min. Each condition was repeated at least three times.

### DNA extraction from basidiospore suspensions

Basidiospore suspensions were prepared by scraping four-week-old PDA-cultured mycelium with sterile distilled water. The concentration was determined using a hemacytometer and then adjusted in sterile water to obtain the desired final concentrations, containing 10^4^, 10^3^, 10^2^, 50, 10, and 0 basidiospores per 1 μL. DNA was extracted from these basidiospore suspensions in order to evaluate the effectiveness of the LAMP assay in detecting basidiospores of *H. annosum*.

### LAMP assay on wood samples

In order to evaluate the ability of LAMP to detect *H. annosum* in wood, trials were conducted following Li (2014). *Pinus sylvestris*, a cultivar highly susceptible to *Heterobasidion* spp., was selected for this experiment. Six pieces of *P. sylvestris* almost 20 cm long and 30–35 cm in diameter were prepared for this assay. Each piece was disinfected with 75% ethanol, wiped with distilled water, and air dried. Three pieces of wood were inoculated with strains of *H. annosum*; the other three were sprayed only with sterile distilled water. The pieces of wood were incubated in a partially darkened room for five weeks, with the surface kept moist during this period. DNA was extracted from each piece of wood and stored at –80°C until used.

## Results

### Design of LAMP primers

**Figure 1:**
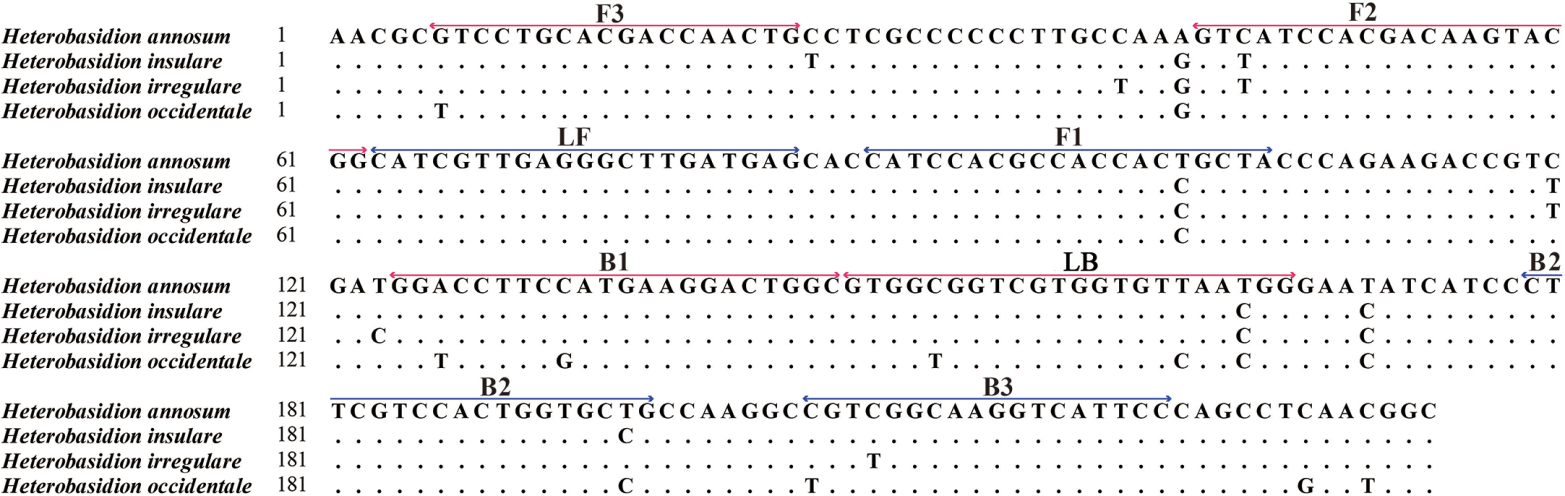
Genomic alignment between *Heterobasidion annosum* and *H. insulare*, *H. irregulare*, and *H. occidentale* at the locus selected for design of the LAMP primer sets. The locations of the designed primers (F3–B3) are shown: forward primer FIP includes the F1 and F2 regions; backward primer BIP includes the B1 and B2 regions; and loop primers include the LB and LF regions. (Red color represents the forward primers, and blue color represents the reverse primers).

The primers were designed using the PRIMEREXPLORER V5 software program (http://primerexplorer.jp/lampv5e/index.html) based on GAPDH. Sequences were aligned using MAFFT 7 (https://mafft.cbrc.jp/alignment/server/). Regions conserved among all tested *H. annosum* populations but differentiating between closely related fungal species were selected for the design of LAMP primers (**Figure 1**). We designed a set of four primers to identify six regions of the target DNA, consisting of two inner primers (a forward inner primer FIP and a backward inner primer BIP) and two outer primers (a forward primer F3 and an outer backward primer B3). Additionally, we designed a loop forward primer (LF) and a loop backward primer (LB) to expedite the LAMP reaction. These primers were synthesized by Sangon Biotech. Nineteen sets of primers were designed for *H. annosum*; the set deemed suitable are listed in **Table 2**.

**Table 2:** Primers used in this study.

Target name	Primer name	Primer sequences	Reference
GAPDH	GAPDH-F	YGGTGTCTTCACCACCACYGASSA	Johannesson et al. (2000)
	GAPDH-R	RTANCCCCAYTCRTTRTCRTACCA	
ITS	ITS5	GGAAGTAAAAGTCGTAAC AAG G	White et al. (1990)
	ITS4	TCCTCCGCTTATTGATATGC	
anno-GAPDH-5	BIP	GGACCTTCCATGAAGGACTGGC- CAGCACCAGTGGACGAAG	This study
	FIP	TAGCAGTGGTGGCGTGGATG- GTCATCCACGACAAGTACGG	
	B3	GGAATGACCTTGCCGACG	
	F3	GTCCTGCACGACCAACTG	
	LB	GTGGCGGTCGTGGTGTT	
	LF	CTCATCAAGCCCTCAACGATG	

### Specificity of the LAMP assay

**Figure 2:**
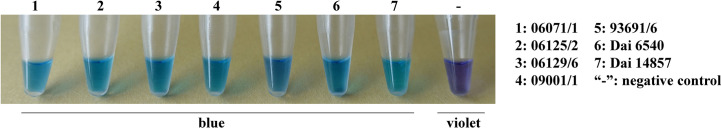
LAMP detection of the GAPDH gene in different isolates of *Heterobasidion annosum*. 1–7: *H. annosum* strains; “-”: negative control.

**Figure 3:**
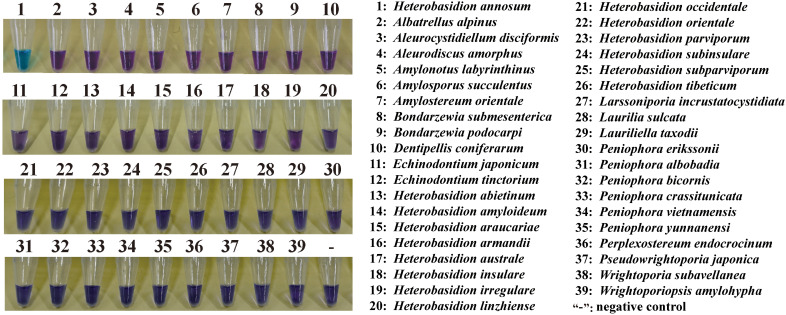
Ability of the LAMP assay to distinguish *H. annosum* from other species in Russulales. “-”: negative control.

DNA from the isolates and specimens of *Heterobasidion* and others, as listed in **Table 1**, were used to validate the specificity of the assay. The LAMP primers were found to detect the species of *H. annosum* accurately. A positive reaction is indicated by a color change from violet to blue in the presence of the HNB indicator (**Figure 2**). GAPDH primers were able to distinguish *H. annosum* from other *Heterobasidion* species, along with fungi commonly detected in wood samples. Based on visual detection using HNB, only samples of *H. annosum* displayed a blue color (**Figure 3**).

### Sensitivity of the LAMP assay

**Figure 4:**
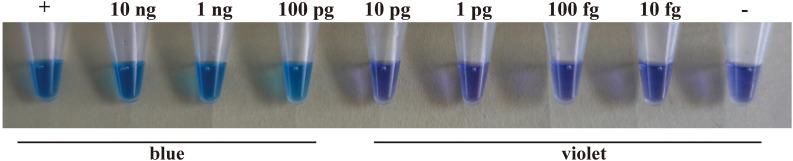
The sensitivity of the LAMP assay using a *H. annosum* s.s. 93961/6 DNA concentration gradient. “+”: positive control; “-”: negative control.

LAMP sensitivity was tested using 10-fold serial dilutions of target genomic DNA prepared with distilled water (10 ng·μL^-1^, 1 ng·μL^-1^, 100 pg·μL^-1^, 10 pg·μL^-1^, 1 pg·μL^-1^, 100 fg μL^-1^, 10 fg·μL^-1^). A Nanodrop spectrophotometer was used to measure DNA concentration. The results showed that a blue color could be detected up to the point where the DNA concentration was as low as 100 pg·μL^-1^. However, the color remained violet when the DNA concentration was reduced further to 10 pg·μL^-1^, 1 pg·μL^-1^, 100 fg μL^-1^, or 10 fg·μL^-1^ (**Figure 4**).

### LAMP assay for basidiospore suspensions

**Figure 5:**
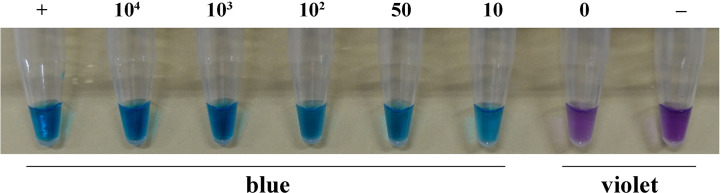
LAMP assay detection of *H. annosum* in basidiospore suspensions containing different numbers of basidiospores. “+”: positive control; “-”: negative control.

The color changed from violet to blue in the suspension of basidiospores from the positive control and other treatments containing 10^4^, 10^3^, 10^2^, 50, or 10 basidiospores per 1 μL. However, in the case of the treatment without basidiospores and the negative control, the color remained violet. This pattern indicated that the LAMP assay could detect the presence of at least ten basidiospores of *H. annosum* per 1 μL in suspension (**Figure 5**).

### Detection in wood

**Figure 6:**
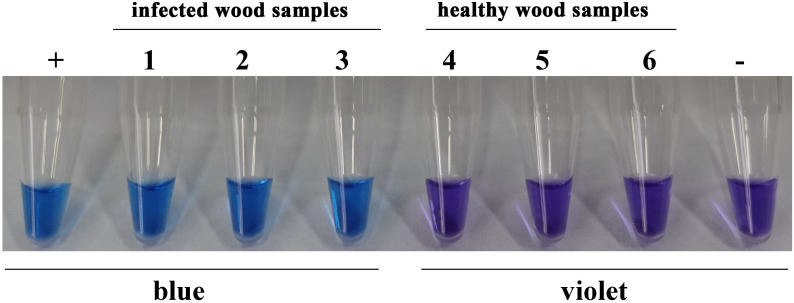
LAMP assay detection of *H. annosum* in wood samples. ”+”: positive control; “-”: negative control.

The LAMP assay was applied to samples of wood infected with *H. annosum*. DNA was extracted from diseased pieces of wood under simulated field conditions; as shown in **Figure 6**, *H. annosum* was successfully detected in diseased wood samples.

## Discussion

Detection of wood decay based on symptoms is relatively difficult. Trees are usually asymptomatic for decades after infection by butt rot, much less for root rot. The external symptoms mostly occur after the sapwood of the tree has decayed (Greig, 1998). Omdal et al. (2004) suggested that aboveground variables can be used as reasonable indicators of root disease. However, the detection of infections caused by slow-growing wood pathogens and with less obvious outer symptoms, such as *H. annosum*, often requires considerable professional knowledge, especially to distinguish closely related species.

Several fragments of genes have been described for detection of species of *Heterobasidion* (Fabritius and Karjalainen, 1993; Kasuga and Mitchelson, 1993; Kasuga et al., 1993; Johannesson and Stenlid, 2003; Linzer et al., 2008; Chen et al., 2014, Chen, 2015; Shamoun et al., 2019; Pellicciaro et al., 2021; Yuan et al., 2021). Although the use of PCR techniques is more successful as a method of detection, it still requires specialized equipment and highly trained personnel, and it is difficult and time-consuming to implement the technique in remote areas and ports. A delay in the identification of wood pathogens causes a major threat to wood production and international trade in timber. We have developed a rapid, specific, and sensitive method of detecting wood decay caused by *H. annosum*, based on GAPDH sequences; furthermore we have evaluated the accuracy of this method in detecting this fungus directly on wood samples. The LAMP method is far more convenient and effective for detecting *H. annosum* in time- and resource- limited conditions. This fungus mostly infects pine (*Pinus* spp.), especially *Pinus sylvestris* (Chen et al., 2015), but also it can be associated with other conifer forests, such as *Abies* sp., *Larix* sp., and *Picea* sp. (Korhonen, 1978). Genetic evidence has confirmed the major significance of stump infection by *H*. *annosum* s.l. (Swedjemark and Stenlid, 1993) in managed forests. The fungus infects freshly cut stumps through the spores and then progresses to the roots, and is able to spread to adjacent trees through root contact (Rishbeth, 1951; Wallis, 1962; Garbelotto and Gonthier, 2013). Thus, our assay may have value during thinning periods in conifer forests.

In general, most PCR amplifications are carried out with a DNA concentration of 20 ng/μL. Conventional PCR amplifications used to detect *Heterobasidion* species are carried out with a DNA concentration of 20 pg/μL (Shamoun et al., 2019). However, the LAMP assay tested in our study was found to detect *H. annosum* with a DNA concentration of 100 pg/μL. With adjustments to the temperature and time, the sensitivity of LAMP assay for detection of *H. annosum* failed to increase. This point necessitates further analysis.

When wood is infected with *H. annosum*, the pathogen may remain active in residual stumps and roots for decades until the next rotation (Rishbeth, 1951; Greig and Pratt, 1976). Significantly, *H. annosum* s.l. grows more quickly in dead trees than living trees. Thus, the method presented here is applicable to the analysis of samples stored for long periods or sent over long distances.

China is one of the biggest timber importers in the world, especially in regard to logs. Conifer logs account for a large proportion of wood imports, and this proportion has climbed from 68.8% to 78.5% since 2017 (Han, 2021). Economic losses to wood decay caused by *H*. *annosum* should not be ignored, and our LAMP assay provides a quarantine tool for reducing such losses through accurate testing of wood samples.

## Data availability statement

The datasets presented in this study can be found in online repositories. The names of the repository/repositories and accession number(s) can be found in the article/**Supplementary Material**.

## Author contributions

Design of the research: ZH-M, YJ, CJ-J; performance of the research: ZH-M, LY; data analysis and interpretation: ZH-M, CJ-J, YJ; collect the materials: DY-C, YY, WC-P; writing and revising the manuscript: ZH-M, CJ-J, YJ, DY-C. All authors contributed to the article and approved the submitted version.

## Supplementary material

SUPPLEMENTARY FIGURE 1 The LAMP assay at different temperatures. The negative control was performed at 63°C for 60 min.

SUPPLEMENTARY FIGURE 2 The LAMP assay at different times. The negative control was performed at 63°C for 60 min.

## References

[B1] Aberg A. Witzell J. Ronnberg J. (2016). Risk of false positives during sampling for heterobasidion annosum s.l. Plant Dis. 100 (1), 175–179. doi: 10.1094/PDIS-03-15-0269-RE 30688580

[B2] Aza A. Kangas A. Gobakken T. Kallio A. M. I. (2021). Effect of root and butt rot uncertainty on optimal harvest schedules and expected incomes at the stand level. Ann. For. Sci. 78 (3). doi: 10.1007/s13595-021-01072-1

[B3] Bendz-Hellgren M. Brandtberg P. O. Johansson M. (1999). Growth rate of *Heterobasidion annosum* in *Picea abies* established on forest land and arable land. Scandinavian J. For. Res. 14 (5), 402–407. doi: 10.1080/02827589950154104

[B4] Caten C. E. Jinks J. L. (1968). Spontaneous variability of single isolates of *Phytophthora infestans.* i. cultural variation. Can. J. Bot. 46, 329–348. doi: 10.1139/b68-055

[B5] Chen J. J. (2015). Taxonomy and phylogeny of Wrightoporia and related genera (Beijing: Beijing Forestry University).

[B7] Chen J. J. Cui B. K. He S. H. Cooper J. A. Barrett M. D. Chen J. L.. (2016). Molecular phylogeny and global diversity of the remarkable genus *Bondarzewia* (Basidiomycota, russulales). Mycologia 108, 697–708. doi: 10.3852/14-216 27091389

[B6] Chen J. J. Cui B. K. Zhou L. W. Dai Y. C. (2015). Phylogeny, divergence time estimation, and biogeography of the genus *Heterobasidion* (Basidiomycota, russulales). Fungal Diversity 71 (1), 185–200. doi: 10.1007/s13225-014-0317-2

[B8] Chen J. J. Korhonen K. Li W. Dai Y. C. (2014). Two new species of the *Heterobasidion insulare* complex based on morphology and molecular data. Mycoscience 55 (4), 289–298. doi: 10.1016/j.myc.2013.11.002

[B9] Dai Y. C. Fan L. F. Chen J. J. Wu C. P. Wu Y. D. Yuan Y. (2021a). Species diversity of conifer pathogen *Heterobasidion* and related quarantine suggestions. Mycosystema 40 (8), 1958–1964. doi: 10.13346/j.mycosystema.210094

[B10] Dai Y. C. Korhonen K. (1999). *Heterobasidion annosum* Group s identified in north-eastern China. Eur. J. For. Pathol. 29 (4), 273–279. doi: 10.1046/j.1439-0329.1999.00153.x

[B11] Dai Y. C. Vainio E. J. Hantula J. Niemelä T. Korhonen K. (2002). Sexuality and intersterility within the *Heterobasidion insulare* complex. Mycological Res. 106 (12), 1435–1448. doi: 10.1017/S0953756202006950

[B12] Dai Y. C. Vainio E. J. Hantula J. Niemelä T. Korhonen K. (2003). Investigations on heterobasidion annosum s. lat. in central and eastern Asia with the aid of mating tests and DNA fingerprinting. For. Pathol. 33 (5), 269–286. doi: 10.1046/j.1439-0329.2003.00328.x

[B13] Dai Y. C. Yang Z. L. Cui B. K. Wu G. Yuan H. S. Zhou L. W.. (2021b). Diversity and systematics of the important macrofungi in Chinese forests. Mycosystema 40 (4), 770–805. doi: 10.13346/j.mycosystema.210036

[B14] Duan Y. B. Ge C. Y. Zhang X. K. Wang J. X. Zhou M. G. (2014). A rapid detection method for the plant pathogen *sclerotinia sclerotiorum* based on loop-mediated isothermal amplification (LAMP). Australas. Plant Pathol. 43 (1), 61–66. doi: 10.1007/s13313-013-0239-6

[B15] Edmonds R. L. Shaw D. C. Hsiang T. Driver C. H. (1989). Impact of precommercial thinning on development of *Heterobasidion annosum* in Western hemlock. USDA For. Service Gen. Tech. Rep., 85–94.

[B16] Enicks D. A. Bomberger R. A. Amiri A. (2020). Development of a portable LAMP assay for detection of *Neofabraea perennans* in commercial apple fruit. Plant Dis. 104 (9), 2346–2353. doi: 10.1094/PDIS-09-19-2036-RE 32697656

[B17] Fabritius A. L. Karjalainen R. (1993). Variation in *Heterobasidion annosum* detected by random amplified polymorphic DNAs. Eur. J. For. Pathol. 23 (4), 193–200. doi: 10.1111/j.1439-0329.1993.tb01338.x

[B18] Gams W. Hoekstra E. S. Aptroot A. (1998). CBS Course on mycology (Monterey, CA, U.S: Centraalbureau voor Schimmelcultures, AG Baarn, the Netherlands).

[B19] Garbelotto M. Gonthier P. (2013). Biology, epidemiology, and control of *Heterobasidion* species worldwide. Annu. Rev. Phytopathol. 51 (1), 39–59. doi: 10.1146/annurev-phyto-082712-102225 23642002

[B20] Goto M. Honda E. Ogura A. Nomoto A. Hanaki K.-I. (2009). Colorimetric detection of loop-mediated isothermal amplification reaction by using hydroxy naphthol blue. BioTechniques 46 (3), 167–172. doi: 10.2144/000113072 19317660

[B21] Greig B. J. W. (1998). Field recognition and diagnosis of heterobasidion annosum. in heterobasidion annosum, biology, ecology, impact and control. Eds. Woodward S. Stenlid J. Karjalainen R. Hüttermann A. (Wallingford: CAB International), 35–41.

[B22] Greig B. J. W. Pratt J. E. (1976). Some observations on the longevity of *Fomes annosus* in conifer stumps. Eur. J. For. Pathol. 6 (4), 250–253. doi: 10.1111/j.1439-0329.1976.tb00533.x

[B23] Han B. (2021). Detailed explanation of china's timber import trends. Construction Sci. Technol. 440 (20), 17–21. doi: 10.16116/j.cnki.jskj.2021.20.003

[B24] Hietala A. Eikenes M. Kvaalen H. Solheim H. Fossdal C. G. (2003). Multiplex real-time PCR for monitoring *Heterobasidion annosum* colonization in Norway spruce clones that differ in disease resistance. Appl. Environ. Microbiol. 69 (8), 4413–4420. doi: 10.1128/AEM.69.8.4413-4420.2003 12902224 PMC169156

[B25] Ioos R. Chrétien P. Perrault J. Jeandel C. Dutech C. Gonthier P.. (2019). Multiplex real-time PCR assays for the detection and identification of *Heterobasidion* species attacking conifers in Europe. Plant Pathol. 68, 1493–1507. doi: 10.1111/ppa.13071

[B26] Johannesson H. Stenlid J. (2003). Molecular markers reveal genetic isolation and phylogeography of the s and f intersterility group of the wood-decay fungus *Heterobasidion annosum* . Mol. Phylogenet. Evol. 29 (1), 94–101. doi: 10.1016/S1055-7903(03)00087-3 12967610

[B1002] Johannesson S. H. Johannesson K. H. P. Stenlid J. (2000). Development of primer sets to amplify fragments of conserved genes for systematic and population studies in the genus Daldinia. Mol. Ecol. 9, 375–378. doi: 10.1046/j.1365-294x.2000.00874-6.x 10736039

[B27] Kasuga T. Mitchelson K. (1993). Determination of the DNA sequence of the 5.8S ribosomal gene of *Heterobasidion annosum* and *Heterobasidion araucariae* . Nucleic Acids Res. 21 (5), 1320. doi: 10.1093/nar/21.5.1320 8464717 PMC309303

[B28] Kasuga T. Woods C. Woodward S. Mitchelson K. (1993). *Heterobasidion annosum* 5.8S ribosomal DNA and internal transcribed spacer sequence: rapid identification of European intersterility groups by ribosomal DNA restriction polymorphism. Curr. Genet. 24 (5), 433–436. doi: 10.1007/BF00351853 7905365

[B29] Kong L. Wang H. B. Wang S. S. Xu P. P. Zhang R. F.. (2020). Rapid detection of potato late blight using a loop-mediated isothermal amplification assay. J. Integr. Agric. 19 (5), 1274–1282. doi: 10.1016/S2095-3119(19)62816-9

[B30] Korhonen K. (1978). Intersterility groups of *Heterobasidion annosum* . Communicationes Instituti Forestalis Fenniae 94, 1–25.

[B31] Korhonen K. Stenlid J. (1998). “Biology of heterobasidion annosum,” in Heterobasidion annosum: biology, ecology, impact and control. Eds. Woodward S. Stenlid J. Karjalainen R. Hüttermann A. (Wallingford: CAB International), pp 43–pp 70.

[B32] Li X. C. (2014). Biological control on heterobasidion parviporum and its decay in China (Beijing: Beijing Forestry University).

[B33] Linzer R. E. Otrosina W. J. Gonthier P. Bruhn J. Laflamme G. Bussières G.. (2008). Inferences on the phylogeography of the fungal pathogen *Heterobasidion annosum*, including evidence of interspecific horizontal genetic transfer and of human-mediated, long-range dispersal. Mol. Phylogenet. Evol. 46 (3), 844–862. doi: 10.1016/j.ympev.2007.12.010 18243021

[B1001] Liu S. L. Zhao Y. Dai Y. C. Nakasone K. He S. H. (2017). Phylogeny and taxonomy of Echinodontium and related genera. Mycologia 109, 1–10. doi: 10.1080/00275514.2017.1369830 29020509

[B34] Mitchelson K. Korhonen K. (1998). “Diagnosis and differentiation of intersterility groups,” in Heterobasidion annosum, biology, ecology, impact and control. Eds. Woodward S. Stenlid J. Karjalainen R. Hüttermann A. (Wallingford: CAB International).

[B35] Mori Y. Nagamine K. Tomita N. Notomi T. (2001). Detection of loop-mediated isothermal amplification reaction by turbidity derived from magnesium pyrophosphate formation. Biochem. Biophys. Res. Commun. 289 (1), 150–154. doi: 10.1006/bbrc.2001.5921 11708792

[B36] Morrison D. J. Johnson A. L. S. (1999). Incidence of *Heterobasidion annosum* in precommercial thinning stumps in coastal British Columbia. Eur. J. For. Pathol. 29 (1), 1–16. doi: 10.1046/j.1439-0329.1999.00126.x

[B37] Niemelä T. Korhonen K. (1998). “Taxonomy of the genus Heterobasidion,” in Heterobasidion annosum: biology, ecology, impact and control. Eds. Woodward S. Stenlid J. Karjalainen R. Hüttermann A. (Wallingford: CAB International), 27–33.

[B38] Niu J. H. Jian H. Guo Q. Chen C. L. Wang X. Liu Q.. (2012). Evaluation of loop-mediated isothermal amplification (LAMP) assays based on 5S rDNA-IGS2 regions for detecting *Meloidogyne enterolobii* . Plant Pathol. 61 (4), 809–819. doi: 10.1111/j.1365-3059.2011.02562.x

[B39] Notomi T. Okayama H. Masubuchi H. Yonekawa T. Watanabe K. Amino N.. (2000). Loop-mediated isothermal amplification of DNA. Nucleic Acids Res. 28, 63–64. doi: 10.1093/nar/28.12.e63 PMC102748 10871386

[B40] Oliva J. Mandy M. Wendt L. Elfstrand M. (2017). Quantitative interactions between the biocontrol fungus *Phlebiopsis gigantea*, the forest pathogen *Heterobasidion annosum* and the fungal community inhabiting Norway spruce stumps. For. Ecol. Manage. 402 (10), 253–264. doi: 10.1016/j.foreco.2017.07.046

[B41] Oliva J. Samils N. Johansson U. Bendz-Hellgren M. Stenlid J. (2008). Urea treatment reduced heterobasidion annosum s.l. root rot in *Picea abies* after 15 years. For. Ecol. Manage. 255 (7), 2876–2882. doi: 10.1016/j.foreco.2008.01.063

[B42] Omdal D. W. Shaw C. G. Jacobi W. R. (2004). Symptom expression in conifers infected with *Armillaria ostoyae* and *Heterobasidion annosum* . Can. J. For. Res. 34 (6), 1210–1219. doi: 10.1139/X04-007

[B43] Franco Ortega S. Bustos Lopez M. Nari L. Boonham N. Gullino M. L. Spadaro D. (2019). Rapid detection of *Monilinia fructicola* and *Monilinia laxa* on peaches and nectarines using loop-mediated isothermal amplification. Plant Dis. 103 (9), 2305–2314. doi: 10.1094/PDIS-01-19-0035-RE 31306092

[B44] Parida M. Horioke K. Ishida H. Dash P. K. Saxena P. Jana A.. (2005). Rapid detection and differentiation of dengue virus serotypes by a real-time reverse transcription-loop-mediated isothermal amplification assay. J. Clin. Microbiol. 43 (6), 2895–2903. doi: 10.1128/JCM.43.6.2895-2903.2005 15956414 PMC1151941

[B45] Parida M. M. Santhosh S. R. Dash P. K. Tripathi N. K. Lakshmi V. Mamidi N.. (2007). Rapid and real-time detection of chikungunya virus by reverse transcription loop-mediated isothermal amplification assay. J. Clin. Microbiol. 45 (2), 351–357. doi: 10.1128/JCM.01734-06 17135444 PMC1829040

[B1000] Pellicciaro M. Lione G. Ongaro S. Gonthier P. (2021). Comparative efficacy of state-of-the-art and new biological stump treatments in forests infested by the native and the alien invasive Heterobasidion species present in Europe. Pathogens 10, 1272. doi: 10.3390/pathogens10101272 34684221 PMC8539811

[B46] Petersen M. Ma L. Y. Lu X. N. (2021). Rapid determination of viable but non-culturable *Campylobacter jejuni* in food products by loop-mediated isothermal amplification coupling propidium monoazide treatment. Int. J. Food Microbiol. 351, 109263. doi: 10.1016/j.ijfoodmicro.2021.109263 34116344

[B47] Rishbeth J. (1951). Observations on the biology of *Fomes annosus*, with particular reference to east anglian pine plantations: III. natural and experimental infection of pines, and some factors affecting severity of the disease. Ann. Bot. 15, 221–246. doi: 10.1093/oxfordjournals.aob.a083278

[B48] Santiago T. D. (2021). Portable and label-free quantitative loop-mediated isothermal amplification (LF-qLamp) for reliable COVID-19 diagnostics in three minutes of reaction time: arduino-based detection system assisted by a pH microelectrode. Biosensors 11 (10), 386. doi: 10.3390/bios11100386 34677342 PMC8533988

[B49] Schulze S. (1999). Rapid detection of European *Heterobasidion annosum* intersterility groups and intergroup gene flow using taxon-specific competitive-priming PCR (TSCP-PCR). J. Phytopathol. 147 (2), 125–127. doi: 10.1046/j.1439-0434.1999.147002125.x

[B50] Seifert T. (2007). Simulating the extent of decay caused by *Heterobasidion annosum* s. l. in stems of Norway spruce. For. Ecol. Manage. 248 (1–2), 95–106. doi: 10.1016/j.foreco.2007.02.036

[B51] Shamoun S. F. Hammett C. Sumampong G. Li X. Garbelotto M. (2019). New taxon-specific *Heterobasidion* PCR primers detect and differentiate north American heterobasidion spp. in various substrates and led to the discovery of *Heterobasidion irregulare* in British Columbia, Canada. Pathogens 8 (3), 156. doi: 10.3390/pathogens8030156 31540403 PMC6789490

[B52] Shaw D. C. Edmonds R. L. Littke R. W. Browning J. E. Russel K. W. (1995). Incidence of wetwood and decay in precommercially thinned western hemlock stands. Can. J. For. Res. 25, 1269–1277. doi: 10.1139/x95-140

[B53] Sillo F. Giordano L. Gonthier P. (2017). Fast and specific detection of the invasive forest pathogen *Heterobasidion* irregulare through a loop-mediated isothermal AMPlification (LAMP) assay. For. Pathol. 48 (2), e12396. doi: 10.1111/efp.12396

[B54] Swedjemark G. Stenlid J. (1993). Population dynamics of the root rot fungus *Heterobasidion annosum* following thinning of *Picea abies* . Oikos 66 (2), 247–254. doi: 10.2307/3544811

[B55] Tokuda S. Hattori T. Dai Y. C. Ota Y. Ota Y. (2009). Three species of *Heterobasidion* (Basidiomycota, hericiales), *H. parviporum*, h. orientale sp. nov. and *H. ecrustosum* sp. nov. from East Asia. Mycoscience 50 (3), 190–202. doi: 10.1007/s10267-008-0476-7

[B56] Vettraino A. M. Luchi N. Rizzo D. Pepori A. L. Pecori F. Santini A. (2021). Rapid diagnostics for *Gnomoniopsis smithogilvyi* (syn. *Gnomoniopsis castaneae*) in chestnut nuts: new challenges by using LAMP and real-time PCR methods. AMB Express 11 (1), 1–11. doi: 10.1186/s13568-021-01266-w 34251538 PMC8275702

[B57] Wallis G. W. (1962). Survey of *Fomes annosus* in East anglian pine plantations. Forestry 33, 203–214. doi: 10.1093/forestry/33.2.203

[B58] White T. J. Bruns T. Lee S. Taylor J. (1990). “Amplification and direct sequencing of fungal ribosomal RNA genes for phylogenetics,” in PCR protocols: a guide to methods and applications. Eds. Innis M. A. Gelfand D. H. Sninsky J. J. White T. J. (San Diego: Academic Press), 315–322.

[B59] Woodward S. Stenlid J. Karjalainen R. Hüttermann A. (1998). “Preface,” in Heterobasidion annosum: biology, ecology, impact and control. Eds. Woodward S. Stenlid J. Karjalainen R. Huttermann A. (Wallingford: CAB International), 1–589.

[B60] Wu F. Man X. W. Tohtirjap A. Dai Y. C. (2022). A comparison of polypore funga and species composition in forest ecosystems of China, north America, and Europe. For. Ecosyst. 4, 540–546. doi: 10.1016/j.fecs.2022.100051

[B62] Xu Y. L. Tian Y. He S. H. (2023). Taxonomy and phylogeny of *Peniophora* sensu lato (Russulales, basidiomycota). J. Fungi 9, 93. doi: 10.3390/jof9010093 PMC9865865 36675914

[B61] Yuan Y. Chen J. J. Korhonen K. Francis M. Dai Y. C. (2021). An updated global species diversity and phylogeny in the forest pathogenic genus *Heterobasidion* (Basidiomycota, russulales). Front. Microbiol. 11. doi: 10.3389/fmicb.2020.596393 PMC7817714 33488542

[B63] Zhou H. M. Wu Y. D. Dai Y. C. (2021). A new species of *Albatrellus* sensu stricto (Albatrellaceae, russulales) from China. Phytotaxa 510, 43–52. doi: 10.11646/phytotaxa.510.1.4

