# Supplementary figures and images for: Rapid detection of Heterobasidion annosum using a loop-mediated isothermal amplification assay
Source: Front Cell Infect Microbiol. 2023 Apr 28;13:1134921. doi: 10.3389/fcimb.2023.1134921 (PMC10175688; doi:10.3389/fcimb.2023.1134921)

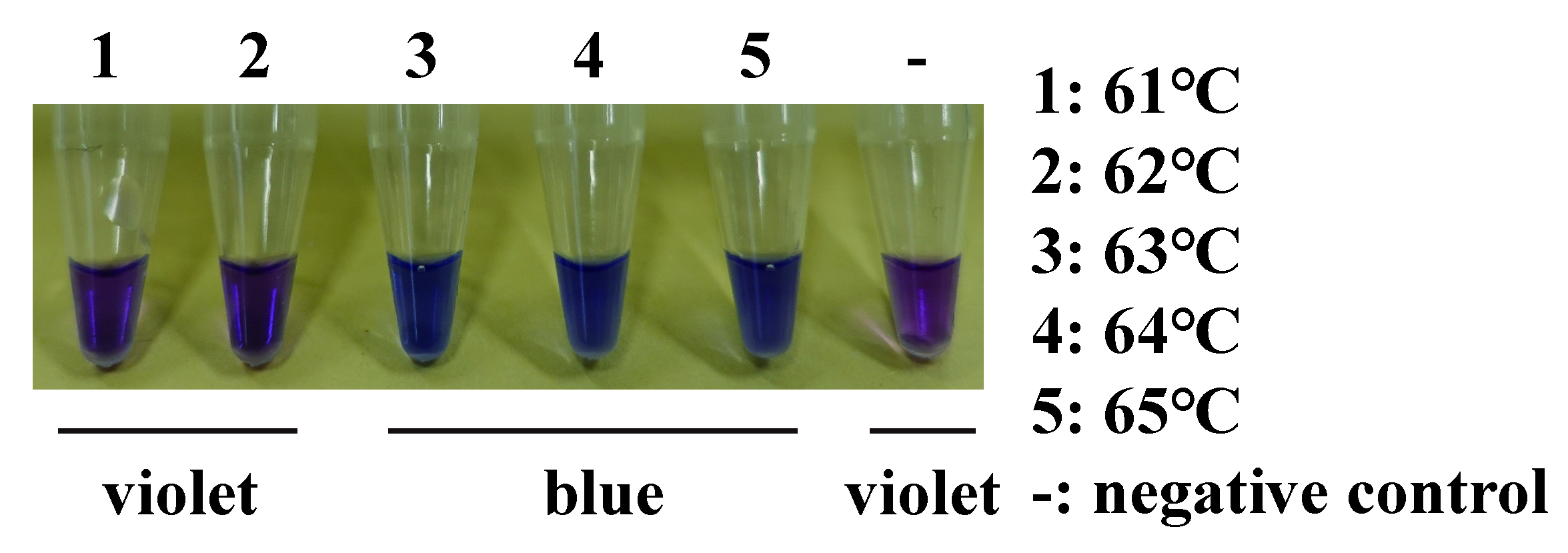

Supplement: SUPPLEMENTARY FIGURE 1 — The LAMP assay at different temperatures. The negative control was performed at 63°C for 60 min. [file Image_1.jpg]

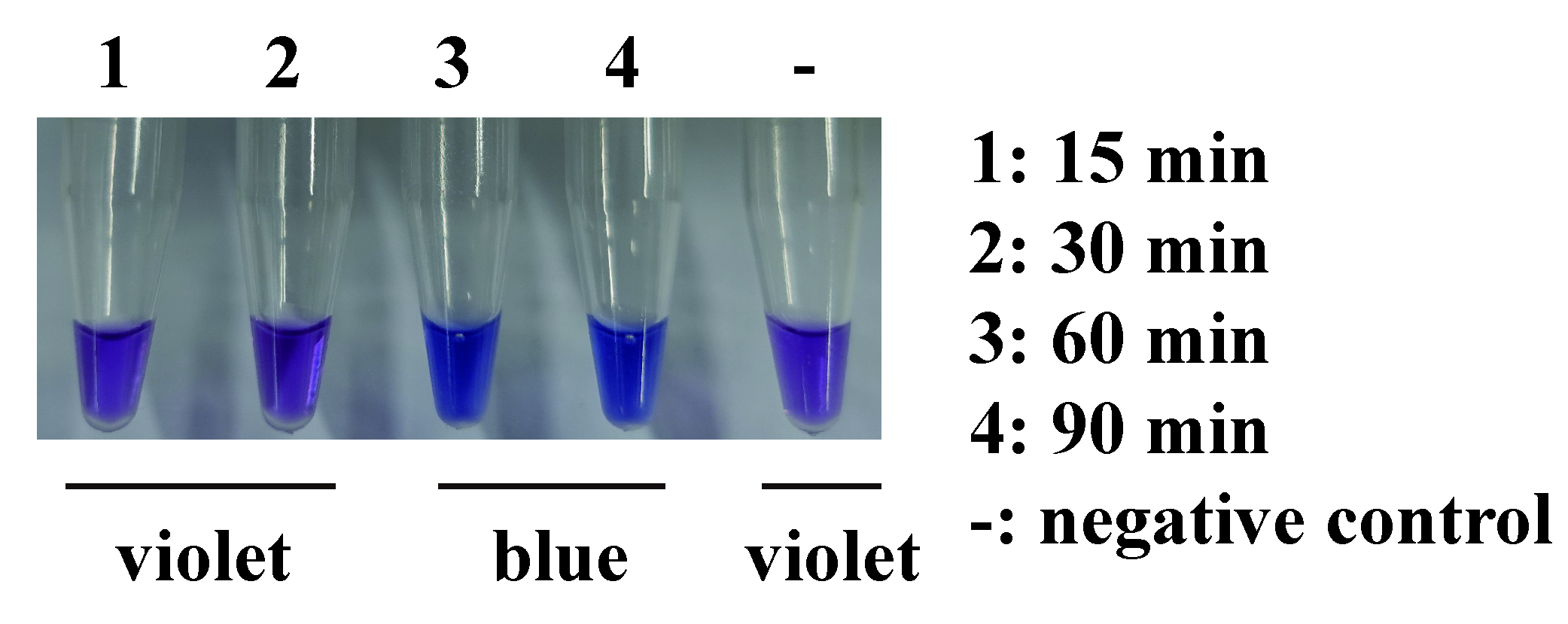

Supplement: SUPPLEMENTARY FIGURE 2 — The LAMP assay at different times. The negative control was performed at 63°C for 60 min. [file Image_2.jpg]
